# Development and pilot validation of a European ethical framework for decision-making in dementia care

**DOI:** 10.1186/s12910-026-01551-y

**Published:** 2026-07-17

**Authors:** Frederik Schou-Juul, Bert Gordijn, Ana Morais, Magda Fonseca, Anthony Scerri, Carsten Hinrichsen, Corinna Porteri, Daniel Sperling, Dervla Kelly, Elzbieta Bobrowicz-Campos, Esra Dogru-Huzmeli, Gabija Jarašiūnaitė-Fedosejeva, Hilde Thygesen, Ieva Stončikaitė, Isabel Machado Alexandre, Joanna Rymaszewska, Kate Irving, Krisztina Zajdó, Linda Johansson, Lucca-Mathilde Thorup Ferm, Olga Riklikiene, Regina McQuillan, Rodrigo Serrat, Rosa Silva, Silke Schicktanz, Therése Bielsten, Yesim Isil Ulman, Sigurd Lauridsen

**Affiliations:** 1 National Institute of Public Health, University of Southern Denmark, Odense, Denmark; 2 Dublin City University, Dublin, Ireland; 3 School of Health and Human Development, University of Évora, Évora, Portugal; 4 Institute of Public Health, University of Porto, Porto, Portugal; 5 Department of Nursing, Faculty of Health Sciences, University of Malta, Msida, Malta; 6 Bioethics Unit, IRCCS Instituto Centro San Giovanni Di Dio Fatebenefratelli, Brescia, Italy; 7 Department of Nursing, University of Haifa, Haifa, Israel; 8 School of Medicine, University of Limerick, Ireland and Health Research Institute, University of Limerick, Limerick, Ireland; 9 Faculty of Psychology and Education Sciences, University of Coimbra, Coimbra, Portugal; 10 Physiotherapy and Rehabilitation Department, Istanbul Gedik University, Istanbul, Türkiye; 11 Department of Psychology, Faculty of Social Sciences, Vytautas Magnus University, Kaunas, Lithuania; 12 Centre for Health and Technology, Faculty for Health and Social Science, University of South-Eastern Norway, Drammen, Norway; 13 Department of Humanities, Pompeu Fabra University, Barcelona, Spain; 14 Instituto Universitário de Lisboa (Iscte) and Instituto de Telecomunicações, Lisbon, Portugal; 15 Department of Clinical Neurosciences, Faculty of Medicine, Wroclaw University of Science and Technology, Wrocław, Poland; 16 School of Nursing, Psychotherapy and Community Health, Dublin City University, Dublin, Ireland; 17 Department of Special Education/Speech-Language Pathology, Apáczai Csere János Faculty of Humanities, Education and Social Sciences, Széchenyi István University, The University of Győr, Győr, Hungary; 18 School of Health and Welfare, Institute of Gerontology, Jönköping University, Jönköping, Sweden; 19 Department of Nursing, Faculty of Nursing, Lithuanian University of Health Sciences, Kaunas, Lithuania; 20 St. Francis Hospice and Beaumont Hospital, Dublin, Ireland; 21 Department of Cognition, Development, and Educational Psychology, Institute of Neurosciences, University of Barcelona, Barcelona, Spain; 22 Nursing School, University of Porto, Porto, Portugal; 23 RISE-Health, Nursing School, University of Porto, Porto, Portugal; 24 Department of Medical Ethics and History of Medicine, University Medical Center Goettingen, Goettingen, Germany; 25 History of Medicine and Ethics Department, Acibadem University School of Medicine, Istanbul, Türkiye

**Keywords:** Dementia care, Ethical decision-making, Clinical ethics, Ethical framework, Content validity, Professional caregivers, Person-centered care, European healthcare

## Abstract

**Background:**

Dementia care involves ethically complex situations across diverse clinical, social care, and policy contexts, yet practice-oriented ethical guidance remains limited. Existing ethical frameworks rarely capture the full complexity of practice across European contexts. This article presents the development and pilot external validation of an Ethical Framework for Decision-Making in Dementia Care, designed for professional caregivers.

**Methods:**

Within COST Action CA21137 (Ethics in Dementia – EDEM), a multidisciplinary European consortium developed the framework through an iterative, deliberative, and consensus-seeking process involving workshops, online meetings, and adaptation of international bioethical norms. An exploratory and preliminary Content Validity Index (CVI) study with external experts assessed the framework’s relevance, clarity, simplicity, and applicability.

**Results:**

The resulting principle-based framework provides non-prescriptive ethical guidance across domains including dignity, autonomy, consent, justice, solidarity, privacy, and public responsibility. The CVI assessment indicated adequate overall content validity (S-CVI/Ave = 0.85), with highest agreement for relevance (0.96) and lowest for applicability (0.75).

**Conclusions:**

The EDEM-Framework offers a structured, exploratory, and practice-oriented contribution to clinical ethics in dementia care. It may strengthen ethical decision-making when supported by appropriate implementation strategies and ethics infrastructures.

**Supplementary Information:**

The online version contains supplementary material available at 10.1186/s12910-026-01551-y.

## Background

Dementia presents one of the most urgent health and societal challenges facing Europe today. The Global Dementia Observatory estimates that over 50 million individuals worldwide currently live with dementia, a figure which is expected to rise to more than 150 million by 2050, in parallel with global population ageing [1, 2]. Dementia care encompasses a broad spectrum of support, including medical management, post-diagnostic interventions, home-based and residential care, psychosocial support, counselling, and assisted living services. Such care is delivered across diverse health and social care contexts, ranging from general practice and community-based services to memory clinics, neurology and geriatric units, psychiatric and palliative care services, and voluntary sector organizations. Given the multifaceted nature of dementia, including progressive cognitive decline, and the diversity of care environments, complex ethical and practical challenges frequently emerge [3–6]. Health and social care professionals must often make difficult decisions in contexts characterized by uncertainty, variable practice standards, and limited guidance [7–10]. In fact, surveys indicate that 91% of community-based professional caregivers report encountering ethical issues, and up to 81% express a need for stronger ethical support in organizing care [11].

Ethical issues arise in situations that undermine, as Côté and Drolet (2021) note, either partially or completely, the respect for at least one desirable ethical principle, and occur as tensions between normatively justified considerations [12]. Such considerations include—but are not limited to—protecting people with dementia and their right to self-determination, recognizing their dignity, monitoring and surveillance and how this may conflict with safety, privacy, medical adherence, or the use and moral weight of advance directives [6, 13].

Existing ethical research has explored numerous complex and interrelated aspects of dementia care, including the disclosure of diagnosis, the right to privacy, consent and assessment of decision-making capacity, and the role of advance care planning or directives in establishing treatment goals, managing intercurrent illnesses, use of assistive technologies and guiding end-of-life care decisions [14–20]. Further areas of inquiry include the ethical implications of access barriers to formal care and the equitable provision of services for people with dementia. Consequently, healthcare professionals are often left with an unmet need for systematic, practice-oriented ethical guidance—a gap compounded by the considerable variation in dementia care pathways across Europe, shaped by differences in healthcare systems, social norms (grounded in a context of age- and dementia- related stigma at the personal, institutional and systemic level) and cultural values, policy contexts, and legislative frameworks.

The purpose of this article is to present the development process of a practice-oriented ethical framework specifically designed to support health and social care professionals within a European context in navigating ethical challenges in dementia care.

## Methods

The Ethical Framework for Decision-Making in Dementia Care (EDEM-Framework) was developed between 2023 and 2025 within the context of the COST Action CA21137: Ethics in Dementia (EDEM). This COST Action comprised a multidisciplinary network and provided broader context for this work. Of this network, a consortium of 26 researchers contributed substantially to the development of the framework. These 26 researchers, who undertook the primary drafting and validation of the framework, had backgrounds in, among others, bioethics and ethics, dementia care and dementia research, nursing, geriatrics and gerontology, psychology and psychosocial sciences, public health and health services research, technology and assistive or rehabilitative technologies, and the health humanities and social sciences (see Table 1).

**Table 1:** Competences of the consortium

Field of competence	Number of researchers*
Bioethics and ethics (including research and medical ethics, law)	10
Dementia care	8
Nursing and nursing science	5
Geriatrics, gerontology and old age psychiatry	4
Psychology and psychosocial sciences	3
Public health, health services and implementation research	5
Technology, AI and assistive/rehabilitative technologies	3
Health humanities and social sciences (e.g. cultural/literary gerontology, sociology)	4

^*^Several researchers contribute expertise to more than one field; totals therefore exceed the number of individual authors

The development process was iterative, characterized by continuous refinement, discussion, and revision of ideas and drafts. Although not all 26 participants attended every meeting on the framework’s content and format, all contributed by commenting on draft versions and key decisions throughout the development process. The process was led by Bert Gordijn, an experienced bioethicist and facilitator who also chaired the working group within the Action responsible for developing the framework. In cases of disagreement regarding content—for example, the wording or relative emphasis of particular principles—or format, such as whether the framework should be organised around perspectives or issues, Gordijn guided deliberations until a version acceptable to all participants was achieved.

The developmental process consisted of four phases comprising five face-to-face workshops with members of the Action: in Belgrade, Serbia (March 2023); Kaunas, Lithuania (September 2023); Valletta, Malta (April 2024); Lisbon, Portugal (September 2024); and Istanbul, Türkiye (September 2025). Between these face-to-face workshops, several online meetings were held to address specific issues that arose and required resolution in order to advance the work and arrive at a final version of the framework. The process was also continuously informed by the ongoing activities of other COST Action EDEM working groups, particularly those focused on examining ethical issues in dementia and identifying existing ethical frameworks relevant to the project.

### Phase 1—Conceptual outlining

The first workshop in Belgrade centered on a review of existing principled ethical frameworks, including the Convention on Human Rights and Biomedicine (1997a) [21], the UNESCO Universal Declaration on Bioethics and Human Rights (2005) [22], the European Code of Conduct for Research Integrity (2017) [23], and the Ethics Guidelines for Trustworthy AI (2019) [24]. Drawing on these sources, the group engaged in structured subgroup discussions to identify the framework’s theoretical underpinnings, level of specificity, and intended target audiences. These discussions were finalized in a joint discussion, which formed the conceptual foundation for subsequent workshops.

At this first workshop, it was also decided that the EDEM-Framework should be directed at a broad range of decision-makers in dementia care, including:

- Policy makers (e.g., government officials, health authorities).
- Management teams (e.g., in nursing homes, daycare centers).
- Dementia care workers (e.g., physicians, nurses, allied health professionals).

The second workshop, held in Kaunas, focused on exploring the potential structure and conceptual orientation of the EDEM-Framework. Participants proposed and discussed several broad, yet interrelated models, organized under four overarching headings:

- A perspective-based framework, addressing ethical issues in dementia care as they typically arise within specific institutions and care settings, such as during diagnosis, home care, long-term care, hospitalization, and within the everyday lifeworld of the person living with dementia.
- A bio-psycho-social-spiritual framework, focusing on ethical challenges as they emerge across physical, psychological and mental, social, and spiritual dimensions of care, and as these issues unfold in interaction with others.
- An issues-based framework organized around well-recognized ethical issues in dementia care that have been discussed in the bioethical literature.
- A stage-based framework, addressing ethical concerns as they manifest across different phases of the dementia trajectory, from mild to severe stages of the condition.

Within this iterative and consensus-seeking design process, after extensive deliberation, the group decided to discard the suggestions under the heading of stage-based framework. Although specific ethical concerns may correspond to particular stages of the disease, patients´ individual disease trajectories vary considerably, making such models imprecise and insufficiently generalized.

### Phase 2—Settling the approach

The remaining three frameworks were further examined during a subsequent workshop in Valletta, where participants initially favored an issues-based approach for its concreteness and immediate relevance to clinical practice. However, at a subsequent meeting in Lisbon, this orientation was reconsidered. It was agreed that while an issues-based framework could appear both practical and pragmatic, it risked becoming overly extensive and potentially misleading, implying that all ethical dilemmas could be exhaustively catalogued and resolved through prescriptive actions. For similar reasons, we discarded the bio-psycho-social-spiritual framework. The underlying idea of this approach is that specific ethical issues can be categorized as biological (e.g., issues regarding diagnostics), psychological (e.g., mental health concerns such as depression), social (e.g., social networks and support structures), or spiritual (e.g., personal faith and deeply held moral commitments). However, we ultimately found it unpersuasive that an exhaustive and meaningful list of ethical issues could be constructed within these four categories.

The perspective-based framework was also rejected. After reflection and deliberation, we concluded that the differences among the relevant professional perspectives in dementia care such as those of social workers, nurses, and medical doctors—were not substantial enough to warrant fundamentally different ethical perspectives. Consequently, due to the shortcomings of the frameworks considered, the author group decided instead to adopt a principle-based organisation of the framework, aligning with other major ethical frameworks such as the Convention on Human Rights and Biomedicine (1997a) [21] and the UNESCO Universal Declaration on Bioethics and Human Rights (2005) [22]. The reasons for adopting this approach were threefold. First, it guarantees a close alignment with the above-mentioned Convention and Declaration, which are both well known and have been widely endorsed internationally. Second, a framework organised around relevant ethical principles operates independently of any sometimes questionable and fundamentally superfluous assumptions about issues, perspectives, dimensions of care or stages of dementia. Third, it provides a practical framework that can be used as a heuristic for caregivers intent on discussing any ethical issue they encounter, as it helps them find the ethical principles at stake and balance those that are conflicting, taking into account the relevant aspects of the case at hand. The 16 principles of the framework have been organised into five clusters based on similarities, each with its own subheading. This makes it easier to quickly find relevant principles when operating in time- and resource-constrained settings.

### Phase 3—Formulation and refinement

The adopted principle-based framework was developed by adapting and refining existing sets of bioethical norms, particularly those articulated in the UNESCO Universal Declaration on Bioethics and Human Rights (2005) [22]. Sections of the Declaration deemed irrelevant to dementia care were omitted. At the same time, the remaining principles were adjusted to better reflect the lived realities and specific ethical challenges encountered in dementia care contexts. Additional insights were drawn from the Nuffield Council on Bioethics Report on Dementia (2009) [5], and key scholarly contributions addressing ethical issues in dementia care [4, 6].

The framework underwent a final comprehensive review during a workshop in Istanbul in 2025, during which discussions also focused on dissemination and implementation strategies across European dementia care settings.

### Phase 4—External validation

The refined framework was externally validated by European experts in dementia care and ethics using a pilot Content Validity Index (CVI) study [25, 26]. CVI is a well-established method for assessing expert agreement on the relevance and quality of items in newly developed frameworks, with modified kappa statistics (κ*) used to adjust for chance agreement in small expert panels [25]. The validation assessed the relevance, clarity, simplicity, and applicability of each ethical principle and aimed to strengthen the methodological transparency and robustness of the framework.

Experts were purposively selected by members of the COST Action, in accordance with methodological recommendations for content validation studies [25, 27]. Inclusion criteria included more than five years of professional experience in dementia care, current professional activity as educators of healthcare professionals, managers of care services, or direct care providers, and demonstrated expertise in addressing ethical challenges through practice, education, policy, or research. Fourteen experts were invited by email and provided with study information.

Five experts completed an online questionnaire (in *Supplementary file*) rating each principle on a four-point Likert scale with respect to its relevance, clarity, simplicity and applicability. The experts were also invited to provide sociodemographic data and qualitative feedback through open-ended questions (including comments, suggestions for improvement, opinion concerning the completeness of each principle, the utility in the daily practice and the motivation to use and disseminate the framework among peers). Responses were pseudo-anonymized and analyzed in compliance with the European General Data Protection Regulation. The study was approved by the Ethical Committee of University of Evora, Portugal (24 October 2025).

The purpose of the pilot external validation was not to reopen the substantive development of the framework’s principles, but to obtain a structured preliminary external appraisal of their relevance, clarity, simplicity, and applicability following the main iterative, deliberative, and consensus-seeking development process. Accordingly, the external experts contributed evaluative feedback — including comments on wording and practical interpretation — rather than acting as co-developers of the framework, and no principle was revised solely on the basis of CVI scores or isolated disagreement.

## Results

### Pilot external validation

Responses to the questionnaire of the pilot external validation were collected between 4 December 2025 and 4 January 2026. Experts´ sociodemographic characteristics are described in Table 2.

**Table 2:** Sociodemographic characteristics of participants

**Experts**	**Country of residence**	**Sex**	**Years of professional experience in the field of dementia**	**Expertise**
E1	Portugal	Female	> 10	Neuropsychology
E2	Poland	Female	> 10	Patient advocacy
E3	Netherlands	Male	> 10	Elder law
E4	Belgium	Male	> 10	Biomedical ethics
E5	Italy	Male	> 10	Neurology and Bioethics

Content validity was evaluated using CVI at both item (I-CVI) and scale level (S-CVI/Ave), following the recommendations of Polit et al., 2007 [25].

The framework demonstrated adequate overall content validity, with a global S-CVI/Ave of 0.85 (Table 3). This value meets commonly cited thresholds for acceptable scale-level content validity (S-CVI/Ave ≥ 0.80) [25]. Among the evaluated criteria, relevance yielded the highest S-CVI/Ave (0.96), followed by clarity (0.86) and simplicity (0.83). Applicability showed the lowest S-CVI/Ave (0.75). At the item level, several principles achieved complete expert agreement (I-CVI = 1.00). Principle 16, Public *responsibility,* reached an I-CVI of 1.00 across all four criteria, indicating unanimous expert ratings for relevance, clarity, simplicity, and applicability.

**Table 3:** Content validity indices by criterion

Criterion	S-CVI/Ave	Mean κ*
Relevance	0.96	0.92
Clarity	0.86	0.78
Simplicity	0.83	0.75
Applicability	0.75	0.70
**Overall**	**0.85**	**0.79**

S-CVI/Ave: Scale level-Content Validity Index/Average

κ*: Modified kappa statistics

To adjust for chance agreement, κ* was calculated. The mean κ* value was 0.79 (Table 3), corresponding to excellent agreement beyond chance according to published benchmarks [25]. Most principles demonstrated κ* values within the good to excellent range. Lower scores were primarily observed for the applicability criterion. These findings are reported descriptively and are further addressed in the Discussion section.

Responses to the open-ended questions were generally positive. Generally, experts considered the framework quite useful to their daily practice, its content quite sufficient to cover the demanded ethical requirements of dementia care and they feel motivated to use and disseminate it among their peers.

## The EDEM-Framework

### Ethical framework for decision-making in dementia care

#### General provisions

**Scope**

This framework contains ethical principles designed to help formal caregivers of persons with dementia make ethically sound decisions when they encounter ethical problems. ‘Formal caregivers’ in dementia care are understood to be healthcare professionals who are paid to provide specialist care and support to people with dementia. They are usually employed by organizations such as healthcare facilities, home care agencies, social care providers and specialized memory care centers.

#### Objectives

The objectives of this framework are: (a) to provide ethical principles—i.e. assertions about values, virtues, duties or entitlements—to guide formal caregivers in addressing the ethical problems they encounter; (b) to promote respect for human dignity and safeguard the human rights of people with dementia in accordance with international human rights law; (c) to promote informed and ethical deliberation and decision-making among formal caregivers to ensure the highest quality of care for people with dementia.

#### Ethical deliberation and decision making

Ethical problems arise in situations where the right course of action is uncertain. This uncertainty may arise from: 1) ambiguity regarding which ethical principles are relevant to the situation, or 2) conflicts between applicable ethical principles, making it challenging to prioritize one over the other. In situations of ambiguity, this framework can help to identify the relevant ethical principles. However, the framework does not provide a fixed ranking of principles. Therefore, caregivers will have to balance conflicting principles themselves considering the specific problem context. In some situations, they may have clear intuitions about the priority of one principle over another. In other, more complex situations, a structured form of team deliberation and decision-making may be helpful. Various methods and procedures have been developed and described in literature. They usually include a schedule of deliberation steps. A simple and useful scheme to guide the discussion is the following: 1) Clearly identify and formulate the ethical problem; 2) Collect the facts relevant to the problem; 3) Identify and discuss the ethical principles that are at play; and 4) Make a decision on how to deal with the problem based on the preceding analysis. It may be helpful to have a moderator to guide such a team deliberation.

#### The role of ethics committees

Multidisciplinary ethics committees should be established, promoted and supported at the appropriate level in dementia care residences in order to: (a) raise awareness and organize appropriate ethics training for formal caregivers, providing them with a basic understanding of ethics and methods of ethical deliberation and decision-making; (b) formulate recommendations and contribute to the development of institutional policies on complex ethical problems that may assist formal caregivers in making ethical decisions; and (c) provide advice on ethical problems that cannot be resolved by the caregivers themselves.

### Ethical principles

#### Fundamental rights and principles

- 1 – Human dignity, human rights and fundamental freedoms

The human dignity of people with dementia is to be fully respected. This involves treating a person in a way that maintains and upholds their value as a human being, as well as respecting who they were and have become. Equally, the human rights and fundamental freedoms of people with dementia are to be fully respected. This includes the right to be free from torture and other cruel, violent, inhumane or degrading treatment or punishment, and the right to freedom of movement to ensure access to communities. Limitations of these freedoms such as the use of physical or chemical restraint, neglect, or limited access to or segregated recreational activities may remove the agency of people with dementia.

- 2—Holistic and personalized care

Care for people with dementia should be holistic—addressing physical, emotional, social, and spiritual needs—and personalized, respecting their autonomy, preferences, and values to enhance their well-being and quality of life.

- 3 – Proportionality of benefits and harms

In healthcare interventions and services, the well-being of the person with dementia must take precedence over societal or scientific interests. Any potential harm to them should be minimized, ensuring proportionality of benefits and harms in all decisions.

- 4 – Respect for autonomy and individual responsibility

The autonomy of individuals to make and take responsibility for their decisions, while respecting others’ autonomy, must be upheld and promoted. In dementia care, the progressive cognitive decline reduces autonomy, necessitating tailored measures to protect the rights and interests of those affected. Promoting autonomy means fostering relationships, offering meaningful choices in daily activities, and supporting individuals in maintaining their identity and values, even as decision-making abilities decline. When individuals can no longer exercise autonomy, trusted people may need to make decisions on their behalf, guided by advance directives, known preferences, or consistent behaviors, in line with their rights and dignity.

#### Informed consent and capacity

##### Consent

Consent is a process based on a trusted relationship between patient and health and social care professionals. Any preventive, diagnostic and therapeutic intervention must be carried out with the prior, free and informed consent of the person with dementia. This consent must be based on adequate and truthful information provided within comprehensible communication and should be obtained in the manner and by the most appropriate means to people with dementia, who can also involve family members and/or trusted persons to make joint decisions.

The ability of the person with dementia to give (and withdraw) consent should not be underestimated and should be carefully assessed as the diagnosis of dementia and/or neuropsychological tests performance do not identify competence, which is task-specific and may vary over time.

Advance care planning should be promoted for people with dementia to communicate their own idea of what they value and to communicate their choices before the severe stage of the disease prevents it.

##### Persons without the capacity to consent

Lack of capacity to give consent cannot be a reason to deny people with dementia the best possible health and social intervention. In accordance with domestic law, special protection is to be given to people with dementia who do not have the capacity to consent.

Decisions for health and social intervention should be aligned with what the individual would have wanted based on their advance directives, past statements, or consistent behaviors and beliefs; when an individual’s preferences are unknown decisions should be taken in accordance with their best interests.

The person with dementia should be involved to the greatest extent possible in the decision-making process; their assent should be sought whenever possible and their refusal should be considered.

#### Respect for individuality and privacy

##### Respect for individual identity, personal integrity and human vulnerability

In applying and advancing scientific knowledge, clinical practice and technologies, individual identity, personal integrity and human vulnerability should be considered.

The person with dementia remains the same, equally valued, person throughout the course of their illness, regardless of the extent of the changes in their cognitive and other functions: their identity should be recognized and their personal integrity respected.

Respect for personal integrity is respect for the physical and mental life of the individual, for their own understanding of their life and illness and for their life story.

The person with dementia can be in a position of increased vulnerability, as their dignity or autonomy or integrity may be threatened: they should be protected to the necessary extent and receive adequate support to enable them to realize their potential.

##### Privacy and confidentiality

Respect for personal integrity entails that the privacy of people with dementia and the confidentiality of their personal information should be respected. Such information should not be used or disclosed for purposes other than those for which it was collected or consented to, consistent with domestic and international law.

#### Equality, inclusion, and non-discrimination

##### Equality, justice, and equity

The inherent dignity and fundamental rights of all human beings must be upheld in dementia care, in a just and equitable way. *Equality* ensures that all people with dementia are treated with equal respect, and access to care and support. *Justice* requires fair access to care and resources, while *equity* demands that care be tailored to the unique needs of each person, recognizing their specific circumstances and challenges.

##### Solidarity

Solidarity in dementia care involves a collective responsibility to support people with dementia. It calls for fostering inclusive and adapted environments and attitudes that combat stigma, while promoting the dignity and rights of those affected by dementia.

##### Non-discrimination and non-stigmatization

People with dementia must be protected from all forms of discrimination and stigma. *Discrimination* arises from unequal access to healthcare, social services, or employment based on characteristics such as race, ethnicity, gender, age, disability, sexual orientation, or other personal attributes. *Stigmatization* stems from biases, negative stereotypes, and misconceptions that marginalize and dehumanize people with dementia. Both practices undermine human dignity, rights, and freedoms, creating barriers to essential care and support.

##### Respect for cultural diversity and pluralism

Dementia care must respect cultural diversity and pluralism by recognizing and valuing the distinct cultural, linguistic, and social backgrounds of all people with dementia. Care approaches should be sensitive to these differences while ensuring that cultural practices do not compromise human dignity, rights, or freedoms. While cultural diversity enriches care, all practices must align with the overarching principles of dignity, rights, and equity, enhancing—rather than limiting—the quality and accessibility of care.

#### Rights and responsibilities

##### Citizens’ rights, political and democratic participation

According to their capacity, people with dementia have the right to participate in political processes and contribute to decisions that affect them, at both the national and local levels. All people with dementia have the right to freedom of expression.

##### Right to health and social care

People living with dementia have rights to equitable access to high quality medical and social care, opportunities to maintain social health, be integrated in the social community and continue participation in social life to the desired. Interactions with people with dementia should be grounded in empirical evidence, relevant professional standards, cultural competence and communication skills to ensure the highest standards of care.

##### Training and skills development

Professionals involved in dementia care must receive adequate training and education to provide high-quality care*.* The quality of care provided shall be measured by adequate indicators and monitored regularly, being a continuous dynamic and iterative process of planning, doing, checking and acting.

##### Public responsibility

Public health professionals have the responsibility to support caregivers and relatives in their roles through education and respite from their duties and to educate and inform the general public of the emerging risk reduction strategies to reduce dementia prevalence and incidence.

## Strategies for dissemination and implementation of the EDEM-Framework

The EDEM-Framework is, first and foremost, designed as a deliberative instrument: a structured tool to support team-based ethical reflection on concrete dilemmas in dementia care. Its effective use as a resource for ethical deliberation presupposes a receptive professional, organizational, and political environment in which it is implemented. Additionally, the value and uptake of the framework in dementia care depend not only on its internal normative coherence, but also on the extent to which the wider policy and governance context enables its meaningful application and sustained implementation.

While the EDEM-Framework is primarily addressed to professional caregivers, its use should be understood as compatible with, and supportive of, the participation of people with dementia in decisions affecting them. In this respect, the framework should not be applied as a substitute for the person’s voice, but rather as a deliberative aid for professionals seeking to interpret and respond to ethically complex situations in ways that remain attentive to the person’s will, preferences, values, assent, and refusal. This is particularly important in relation to consent, autonomy, and decision-making support, where ethical practice in dementia care requires not only professional judgement but also sustained efforts to involve the person with dementia to the greatest extent possible.

These considerations also point to a broader implementation issue. The EDEM-Framework is unlikely to influence dementia care practice unless attention is given to how it is introduced, supported, adapted, and sustained within professional and organisational settings [28–30]. In this respect, it is useful to distinguish between the framework itself and the strategies used to disseminate and implement it. The EDEM-Framework is the object of implementation; dissemination, training, organisational embedding, facilitation, and evaluation are means through which it may become usable in everyday ethical deliberation. The following section therefore outlines practical considerations for disseminating, implementing, and evaluating the framework across different dementia care and ethics-infrastructure contexts.

Drawing on Wandersman et al.’s (2008) Interactive Systems Framework (ISF), implementation of the EDEM-framework can be understood as engaging three interrelated systems: the Synthesis and Translation System, which converts abstract ethical principles into usable tools and materials; the Support System, which includes ethics committees and training programs that build caregivers' capacity; and the Delivery System, where caregivers apply ethical principles/framework in practice [31]. Each system must be activated to bridge the gap between principle and practice.

### Dissemination

Dissemination of the framework is a prerequisite for its implementation. Without deliberate efforts to make the framework visible, intelligible, and accessible to different stakeholder groups, its potential to inform ethical dementia care will remain largely unrealized. As situated within Wandersman et al.’s (2008) Synthesis and Translation System, dissemination involves translating abstract ethical principles into applicable, context-relevant materials and ensuring they reach the appropriate audience [31]. For professional caregivers, this requires more than passive distribution; dissemination must be strategic, targeted, and adapted to their roles, settings, and informational needs. It is the first step in building awareness and shared understanding of the framework’s purpose and use.

According to Scott et al.’s (2024) consolidated framework for disseminating research, the first steps are the targeted identification and engagement of dissemination partners [32]. For this ethical framework in dementia care, we have a threefold audience: policy makers, management teams in dementia services and dementia care workers (as highlighted in Development and validation). Policy makers should be reached through policy briefs that illustrate how the framework can inform regulatory instruments and broader structural approaches to dementia care, including national dementia strategies, which vary greatly in terms of breadth and ethical focus throughout Europe. For management teams, dissemination should occur via presentations at practice-oriented conferences and through collaboration with national non-governmental organizations (NGOs), such as national Alzheimer associations, which already maintain established networks among service managers. Finally, for dementia care workers, dissemination strategies could include integrating the EDEM-Framework in education efforts and materials and relevant conferences.

Dissemination strategies should be informed by likely barriers and enablers [32]. Anticipated barriers include competing policy, managerial, and clinical priorities, as well as resource constraints. Potential enablers include established dementia NGOs, growing political recognition of ethical challenges in dementia care, and documented needs among health and social care professionals for structured ethical support. These conditions may support dissemination and facilitate subsequent uptake of the EDEM-Framework across European contexts.

### Implementation

Implementation of the EDEM-Framework in dementia care may be pursued along two mutually reinforcing tracks. The first and primary track is the deliberative practice track, which embeds the framework in everyday ethical decision‑making by individual professional caregivers or teams. The second track concerns the wider implementation across European contexts, which supports translation of the framework across heterogeneous health and social care systems into deliberative practice. Taken together, these aspects of implementation involve interrelated micro‑level decision-making, meso‑level organizational routines, and macro‑level system and policy structures.

#### Implementing the framework in practice

At the micro- and meso- levels, the primary mode of deployment of the EDEM-Framework in dementia care includes ethical case deliberation in clinical practice. Rather than specifying prescriptive resolutions, the framework is designed to structure moral reasoning in such situations, by offering a way to identify, analyze, and deliberate on ethical dimensions of practice. It is intended for use when caregivers confront situations in which the ethical considerations are unclear, difficult to articulate or manage, or in tension with one another. Many such situations concern how respect for autonomy should be understood and sustained under conditions of cognitive decline. In this context, support for autonomy may require not only interpersonal and relational support, but also attention to cognitive and communicative strategies that enable people with dementia to understand options, express preferences, and participate in decisions to the greatest extent possible. In this respect, the framework should not be viewed as a substitute for communicative or cognitive support strategies, but as a deliberative aid that is compatible with their use and may help structure professional reflection on their ethical relevance and appropriate application.

The principles articulated in the framework have moral content, but they must be weighed against one another during collective decision-making [33]. As ethical issues become more complex, it is rarely sufficient for a single professional to grasp all relevant moral dimensions; instead, health and social care teams can convene ethics reflection meetings to analyze difficult cases. These may take the form of informal ethics rounds or more formal moral case deliberation sessions led by trained facilitators, which have been shown to foster changes in practice, particularly among professional caregivers working in inter-professional constellations [34]. Such sessions may be inspired by existing methods of ward-based ethical decision-making and clinical ethical reasoning described in the literature, which typically involve a structured sequence of identifying the ethical problem, clarifying relevant facts, discussing the values or principles at stake, and arriving at a reasoned course of action [33, 35, 36]. Apart from the framework as a shared point of reference, the principles help to organize the conversation, make value conflicts explicit, and support the development of reasonable ethical decisions. In such sessions, the framework lends itself to team-based deliberation.

The EDEM-Framework may also guide deliberation within more formal structures such as ethics committees or management teams in dementia care settings. Ethics committees, nursing home managers, or similar bodies can use it both to deliberate on particularly challenging clinical cases and to develop local ethical guidelines for recurrent issues. By informing institutional policies, e.g., on restraint, surveillance, technology use and consent, the framework may help to ensure that caregivers receive advice on ethical issues in the care of people with dementia.

#### Implementation across European contexts

At the macro level, which underpins and conditions implementation at the micro and meso levels, governments and relevant professional bodies establish the regulatory, policy, and organizational frameworks that shape ethical practice across European health and social systems. As Howlett (2018) argues, on this level, implementation may be argued to unfold through a series of *critical junctures* in the wider policy process [37]. In this perspective, implementation depends not only on the quality of the framework, but also on the policy windows, actors, resources, and institutional arrangements through which it is introduced. Rather than a neutral technical post-development phase, implementation is a contested arena in which coalitions renegotiate goals, instruments, and resources [37]. For the EDEM-Framework, this means that uptake will depend on whether it can be aligned with existing dementia strategies, ethics infrastructures, professional routines, and organizational capacities. Implementation should therefore be understood as a context-sensitive process in which the framework may be adopted, adapted, or marginalized depending on the institutional conditions in which it is introduced.

Implementation of the EDEM-Framework may potentially take place within European health and social care systems that differ rather substantially in terms of resources, governance, social norms and cultural values, including attitudes toward dementia and existing ethics infrastructures, which may act as either barriers or facilitators of ethical dementia care. In what follows, we reflect on how the framework can be translated into ethical deliberation and organizational practice, and how differences in health system context and ethics infrastructures across Europe may shape its potential uptake and implementation. In fact, to avoid purely generic implementation recommendations at the macro level, it is useful to distinguish between countries according to maturity and reach of what we may distinguish as the *ethics infrastructures* in dementia care. We therefore introduce an analytically derived trichotomy, (I) ethics-embedded systems, (II) ethics-developing systems, and (III) ethics-constrained systems, as a heuristic for structuring the following discussion of implementation recommendations. Such a trichotomy is a pragmatic proxy for implementation readiness and capacity, i.e., encompassing relevant elements for implementation such as institutional stability, availability of training and supervision structures, or the presence of ethics committees or comparable bodies. Across these three systems, key implementation barriers and appropriate strategies are expected to differ (refer to Table 4 for a summary of action plans for each ‘ethics infrastructure’ according to potential barriers and facilitators.):

(I) In ethics-embedded systems, ethics infrastructures in dementia care are relatively mature: National Dementia Strategies (NDSs) are in place and engage with ethical domains of dementia care and may regularly be updated; clinical guidelines explicitly address ethical issues; ethics committees or comparable bodies are established and enjoy normative authority (e.g. in hospitals and, in some cases, in long-term care); and ethics-related content is already integrated into professional education or continuing professional development. In such contexts, ethical reflection is not absent, but it may be fragmented across documents or weakly connected (yet still useful) to everyday practice. In such settings, the primary aim is integration into care practice rather than introduction of ethical frameworks and deliberation. Implementing the EDEM-Framework in such systems first requires effective dissemination strategies, for example leveraging existing synthesis and translations systems. Subsequent steps include aligning its principles and general provisions, including the emphasis on ethical deliberation in practice and the role of ethics committees, with existing policy and governance instruments. This process may also involve navigating the de-implementation of existing, less comprehensive frameworks that are embedded in current institutional routines or policies.
(II) In ethics-developing systems, ethics infrastructures relevant to dementia care are present but more sporadic or fragmented. There may be general health or ageing policies with ethical components, draft NDSs or discussions about content of those, or sector-specific guidelines, but coverage is incomplete, and responsibilities are often split between levels of government and quality varies greatly among public and private providers. Clinical ethics committees may exist in some hospitals or other care institutions, but not in community or long-term care; opportunities for formal ethics training are variable, and there may be substantial regional differences in expertise and resources. In such systems, implementation of the framework may be best advanced through targeted pilots and demonstration models, coupled with structured dissemination. A pragmatic strategy for implementing the EDEM-Framework could be to identify a small number of anchor sites, e.g. memory clinics, or a group of nursing homes, and to introduce the framework there as a basis for ethical case deliberation, local guideline development, and staff training. These sites may serve as laboratories and research sites for adapting the framework to national legal and cultural contexts, generating practice-based examples and materials that can be shared more widely. However, here, engagement with partners and network-building is crucial. Professional associations, Alzheimer and dementia organizations, and academic centers can form national or regional working groups acting as synthesis and translation systems that adopt the framework as a common reference point. These networks can organize workshops, develop training modules, and produce practice notes or case collections that illustrate how the framework can be used in different settings (acute care, home care, long-term care). Where partial dementia strategies exist, the framework can be used to inform revisions and help make the ethical dimension of those policies more explicit and coherent.
(III) In ethics-constrained systems, formal ethics infrastructures in dementia care are limited. In these systems, NDSs are absent or only in early fragmented development; ethics committees are rare or outside tertiary (likely private) hospitals; and many services operate under substantial workforce and funding pressures. For caregivers working under such conditions, ethical reflection must be framed not as an additional burden, but as a practical support for navigating the difficult situations they face while trying to make it through the day. Ethical issues are no less present in these contexts, but opportunities for systematic ethical reflection and governance are fewer, and the focus of policy is often on securing basic access to diagnosis and care. Here, implementation of the EDEM-Framework is best conceived as a program of capacity-building, literacy-building, and legitimacy-building. At the macro level in ethics-constrained systems, the EDEM-Framework can serve as a reference document for those involved in drafting the first dementia strategies or related policies. Even if the framework cannot be implemented comprehensively, its principles can inform the ethical vocabulary of emerging policies, contributing to relevant considerations being articulated from the outset. At the same time, efforts should focus on developing ethical literacy among formal caregivers and managers. Building legitimacy is equally important. In systems where ethics infrastructure is constrained, ethical reflection may be perceived as a luxury or as external criticism. Positioning the framework as a practical tool that helps caregivers navigate difficult situations, rather than as a mechanism of control, may help mitigate this perception.

**Table 4:** Summary of action plans for each ‘ethics infrastructure’ according to potential barrier/facilitators

Barrier/Facilitator	Actions in Ethics-Embedded Systems	Actions in Ethics-Developing Systems	Actions in Ethics-Constrained Systems
Ethics infrastructure varies greatly across countries	Integrate EDEM into existing ethics committees, update policies to incorporate principles, and align with established national dementia strategies	Pilot EDEM in selected sites, develop national working groups, create practice examples, and begin harmonizing ethics components across services	Use EDEM as a foundational reference in emerging policies; build basic ethics awareness and introduce simple deliberation tools
Ethical deliberation processes are inconsistent or fragmented	Strengthen existing deliberation processes; formalize practice moral case deliberation; embed EDEM in supervision structures	Establish ethics rounds in pilot services; train facilitators; produce case collections demonstrating use of EDEM	Introduce short, accessible deliberation steps; provide basic training in identifying ethical problems
Strategic dissemination planning	Develop tailored dissemination materials that align with already established ethics channels	Partner with NGOs and academic institutions to create targeted dissemination campaigns	Provide clear introductory materials and awareness-raising campaigns on ethics in dementia care
Availability of trained facilitators for ethical deliberation	Provide modules to expand facilitator capacity; embed facilitator training into organizational development	Train local moderators; create facilitator networks; integrate EDEM in higher education and continuing education curricula	Offer foundational facilitator competences training; develop simple, low-resource training modules
Availability of resources and presence of competing priorities	Streamline integration by embedding ethics discussions within existing meetings and workflows	Introduce low-resource pilots; integrate ethics into routine documentation; demonstrate efficiency benefits	Use brief, low-burden tools; emphasize practical benefits for daily care challenges
Recognition of ethical challenges and demand for support	Scale up EDEM through national programs; incorporate into policy reforms	Leverage existing enthusiasm to form national ethics networks; co-produce training materials	Frame EDEM as a supportive tool that responds to frontline needs; provide introductory workshops
Existence of ethics committees in community and long-term care settings	Extend committee structures to community care; align roles with EDEM principles	Create small ethics advisory groups in pilot services; evaluate feasibility of expansion	Promote micro-ethics groups or informal ethics meetings; introduce basic committee templates
Cultural diversity and differing norms in dementia care	Produce culturally sensitive adaptations aligned with existing national standards	Co-develop culturally appropriate training materials with regional partners	Provide introductory guidance that recognizes cultural diversity without requiring extensive structures

Future research should focus on the systematic evaluation and monitoring of EDEM implementation across diverse dementia care contexts. Implementation science frameworks, such as RE-AIM, CFIR, or Proctor’s implementation outcomes framework, may offer useful approaches for structuring evaluation efforts across organisational and national settings [38]. Evaluation may include short-term outcomes related to dissemination reach, awareness, and engagement with the framework; mid-term outcomes related to adoption, acceptability, feasibility, fidelity, and integration into routine care practices; and longer-term outcomes related to strengthened respect for human dignity, safeguarding of the human rights of persons with dementia, and strengthened informed ethical deliberation and decision-making among formal caregivers. Mixed-methods approaches combining qualitative and quantitative data may be particularly valuable for understanding contextual barriers, facilitators, and opportunities for iterative refinement of the framework.

## Discussion

The EDEM-Framework and its accompanying dissemination and implementation strategy, as presented in this article, possess several strengths and limitations. First, the framework encompasses sixteen broad, unranked ethical principles that are central to ethical decision-making in dementia care. The breadth and non-hierarchical nature of these principles constitute a key strength, as they enable users to apply the framework as an open analytical lens for identifying and reflecting on relevant ethical conflicts. This flexibility facilitates focused ethical deliberation rather than prescriptive decision-making. However, this openness also presents a limitation: the framework does not provide explicit guidance on how to act when principles come into conflict in specific clinical situations. Some of the principles and methods proposed in the framework may be perceived as controversial by certain users. For example, the framework identifies advance directives and advance care planning as important mechanisms for protecting individuals’ autonomous decision-making regarding their preferred care in later stages of dementia, when they may no longer possess decision-making capacity. However, the ethical appropriateness of advance directives remains contested within bioethics, with some scholars defending their use and others expressing scepticism about their moral authority and practical application [39–41].

We therefore encourage users of the framework to adopt a sensitive and reflective approach to advance directives, viewing them as one important guide to good dementia care among several others. In all cases, attention should remain focused on what constitutes the best care for persons with dementia in their present circumstances and lived experience. Nevertheless, the text of the ethical framework is not cast in marble. The precise wording of the principles may be subject to further refinement or adjustment as the framework is used and discussed in practice. This applies in particular to the current privacy principle, which is largely framed in relation to confidentiality and personal information and may therefore require further conceptual refinement in future iterations of the framework. Privacy in dementia care can also reasonably be interpreted more broadly, encompassing additional decisional, bodily, spatial, physical-local, and relational dimensions. For instance, Buhr and Schweda (2025), writing about privacy in dementia care, distinguish decisional privacy and physical-local privacy in addition to informational privacy [42]. Moreover, some stakeholders may consider the number of principles either excessive or insufficient, depending on their expectations of scope and specificity. An adjacent limitation, concerning the application and implementation of the framework, is the underlying assumption that its users possess sufficient ethical competence to identify the relevant principles in a given case and to apply them appropriately, including recognizing when multiple, potentially conflicting principles are at stake. In fact, efforts to implement the framework may seek to increase the specificity of the principles in relation to dementia care, while also providing concise guidance on how to conduct ethical case deliberation and what constitutes a well-structured, reasonable ethical decision [43]. Moreover, while the framework’s strength lie in offering a comprehensive foundation for ethical reflection among professional caregivers, this focus simultaneously limits its direct applicability to family caregivers and people living with dementia. This limitation should be stated explicitly: the framework was developed primarily by professional and academic stakeholders, and people living with dementia and family carers were not involved as co-producers in the framework development process. Future refinement would therefore benefit from their direct involvement, particularly to strengthen its responsiveness to lived experience and its relevance to supported participation in decision-making, as reflected in Article 12(3) of the UN CRPD [44]. The explicit orientation toward professional users may also reduce its capacity to capture ethical challenges that arise from the dynamic interplay between professional and family caregivers and the person with dementia. Furthermore, another strength is the framework’s compatibility with existing organizational infrastructures, such as ethics committees and patient conferences as well as overall social organization of dementia in the different systems, i.e. mechanisms that operate within many healthcare systems, particularly in more ethics-embedded systems. Nevertheless, its application may be less straightforward in settings where such structures are absent, for example, in less affluent countries or in nursing homes or day care centers that typically lack formal ethical deliberation bodies.

A limitation of the methodological aspects of the development process also warrants explicit acknowledgement. Although the EDEM-Framework was developed through a structured process involving a relatively large group of experts in dementia care and dementia care ethics, informed by a literature review and iterative consensus-seeking discussions, as described in the development section, it did not follow established Delphi procedures.

With regard to the pilot external validation, five experts participated in a CVI assessment of the proposed framework. This step should be understood as an exploratory and complementary validation exercise within a broader, iterative development process involving a substantially larger multidisciplinary expert community through the COST Action. Accordingly, the feedback obtained was considered informative rather than determinative and was not intended to outweigh the extensive collective expertise underpinning the framework’s development.

The small number of external experts means that this stage should be interpreted as a preliminary appraisal of content validity rather than as a representative validation of the broader target population. Although it provided structured external feedback on the relevance, clarity, simplicity, and applicability of the proposed principles, the limited sample size constrains the generalisability of the findings. Future research should therefore extend this work through larger and more diverse expert panels, alongside empirical studies examining the framework’s usability and implementation across different dementia care settings.

CVI was originally developed to assess the content validity of measurement instruments, particularly in relation to relevance and clarity [25–27]. In the present study, its use should therefore be understood as a pilot and exploratory appraisal of content validity, providing a structured external assessment of the framework’s practical comprehensibility and perceived usability. Although its application to ethical frameworks is uncommon, it offers a transparent and systematic approach for documenting expert agreement on dimensions closely related to practical usability. The variation observed across criteria, particularly the comparatively lower scores for applicability, should therefore be interpreted with caution. In the context of ethical frameworks, lower applicability ratings do not necessarily indicate deficiencies in the principles themselves, but may instead reflect the inherent challenges of translating abstract ethical norms into heterogeneous care settings shaped by legal, organisational, cultural, and resource-related constraints [5, 6]. For this reason, principles with CVI values close to, but below, commonly cited thresholds should not be revised solely on the basis of these results, as this could conflate implementation challenges with issues of content validity.

The calculation of κ* further strengthened the interpretation of the CVI findings by adjusting for chance agreement [25]. Overall, the CVI exercise should be regarded as one validation step among several, complementing the framework’s iterative, deliberative, and consensus-seeking development process and pointing to the need for future empirical research on implementation and use. The quantitative findings were also supported by convergent positive qualitative feedback from the participating experts.

Future research should additionally examine the appropriateness, meaningfulness, and feasibility of the framework across diverse dementia care contexts and systems [45, 46]. Such work will be essential to further establish the robustness, transferability, and practical relevance of the EDEM-Framework.

The present framework therefore reflects the combination of iterative, consensus-seeking drafting and structured expert appraisal, while future empirical work on implementation and use will be required to further assess its usability and impact in diverse dementia care contexts.

## Conclusions

This article has presented the Ethical Framework for Decision-Making in Dementia Care (EDEM-Framework), detailing its development, its pilot external validation and its proposed strategies for application, implementation, and dissemination across different dementia care systems. The framework is intended to structure ethical reflection in dementia care and implementation at multiple levels, from team-based case deliberation to the policies and infrastructures that sustain such deliberative practice. By offering ethical vocabulary and a principled, non-prescriptive structure for analyzing ethical dilemmas, it seeks to enhance the quality of ethical decision-making in dementia care.

## Supplementary Information

Supplementary Material 1.

## Data Availability

The datasets generated and/or analyzed during the current study are not publicly available because they contain potentially identifiable expert feedback, but anonymized data are available from the corresponding author on reasonable request.

## Abbreviations

CVI: Content Validity Index
EDEM: Ethics in Dementia
I-CVI: Item-level Content Validity Index
ISF: Interactive Systems Framework
k*: Modified kappa statistic
NDS: National Dementia Strategy
S-CVI/Ave: Scale-level Content Validity Index, average method
