# Supplementary material for: Development and pilot validation of a European ethical framework for decision-making in dementia care
Source: BMC Med Ethics. 2026 Jul 17;27:171. doi: 10.1186/s12910-026-01551-y (PMC13491858; doi:10.1186/s12910-026-01551-y)
Supplement: Supplementary file 1 [file 12910_2026_1551_MOESM1_ESM.pdf]

Supplementary file - *Questionnaire of the pilot external validation*

“Ethical framework for formal caregivers in dementia care

We would like to sincerely thank you for your time and valuable contribution to this study. Your expertise and insights are essential for validating the proposed ethical framework and will play a crucial role in advancing knowledge and practice in the field of ethics and dementia care.

Before beginning the questionnaire, please take into account the following general instructions:

You will be asked to evaluate each item of the framework according to four criteria:

Relevance, Clarity, Simplicity, and Applicability.

Please rate each item using the Likert scales provided.

When appropriate, you may use the open-text fields to comment on items that you believe require clarification, revision, or reformulation.

Your responses should reflect your professional judgment and experience in the field.

The questionnaire should take approximately 20min to complete.

Please ensure that you submit your responses once you have completed all sections.

Thank you once again for your valuable time, expertise, and commitment to improving ethical guidance in dementia care.

* mandatory response

1. What is your sex? *

Select only one option.

Female

Male

Prefer not to say

Other:

2. In which country do you currently reside? *

3. How would you describe your professional experience related to ethics in dementia care or dementia care in general? *

Select only one option.

Less than 1 year

1—5 years

6—10 years

More than 10 years

I do not have professional experience in this area

Ethical framework for formal caregivers in dementia care

An ethical framework for formal caregivers in dementia care is a structured set of principles, values, and guidelines designed to support caregivers in making morally sound decisions while providing care to people living with dementia. Its purpose is to help professionals navigate complex ethical situations — such as issues related to autonomy, dignity, communication, and end-of-life decisions — in a consistent and reflective way.

This framework aims to promote person-centered, compassionate, and ethically responsible care, while also supporting caregivers' moral well-being and professional integrity.

Following the context provided regarding content validity, we kindly request your collaboration in the accurate completion of this scale for each component of this framework, specifically in terms of relevance, clarity, simplicity, and applicability:

Relevance: The degree to which the item is essential and appropriate to the construct.

Clarity: The extent to which the wording and meaning of the item are understandable and unambiguous.

Simplicity: The degree to which the item is concise, straightforward, and avoids unnecessary complexity.

Applicability: The extent to which the item can be applied in practice or is suitable for the intended context.

Responses should be marked with a cross (X) in the corresponding option, with the possibility of including observations when necessary (”Comments”). In addition, we appreciate your opinion concerning the completeness of each principle, the utility of the proposed framework in your daily practice concerning dementia care and your motivation to use and disseminate it among your peers (last three questions).

Fundamental rights and principles

1 - *Human dignity, human rights and fundamental freedoms*

The human dignity of PwD is to be fully respected. This involves treating a person in a way that maintains and upholds their value as a human being, as well as respecting who they were and have become. Equally, the human rights and fundamental freedoms of PwD are to be fully respected. This includes the right to be free from torture and other cruel, violent, inhumane or degrading treatment or punishment, and the right to freedom of movement to ensure access to communities. Limitations of these freedoms such as the use of physical or chemical restraint, neglect, or limited access to or segregated recreational activities may remove the agency of PwD.

4. Relevance *

Select only one option.

Not relevant

Somewhat relevant

Quite relevant

Highly relevant

5. Clarity *

Select only one option.

Not clear at all

Somewhat clear

Quite clear

Highly clear

6. Simplicity *

Select only one option.

Not simple

Somewhat simple

Quite simple

Highly simple

7. Applicability *

Select only one option.

Not applicable

Somewhat applicable

Quite applicable

Highly applicable

8. Comments

Fundamental rights and principles

*2 - **Holistic and personalized care***

Care for PwD should be holistic - addressing physical, emotional, social, and spiritual needs - and personalized, respecting their autonomy, preferences, and values to enhance their well-being and quality of life.

9. Relevance *

Select only one option.

Not relevant

Somewhat relevant

Quite relevant

Highly relevant

10. Clarity *

Select only one option.

Not clear at all

Somewhat clear

Quite clear

Highly clear

11. Simplicity *

Select only one option.

Not simple

Somewhat simple

Quite simple

Highly simple

12. Applicability *

Select only one option.

Not applicable

Somewhat applicable

Quite applicable

Highly applicable

13. Comments

Fundamental rights and principles

3- Proportionality of benefits and harms

In healthcare interventions and services, the well-being of PwD must take precedence over societal or scientific interests. Any potential harm to them should be minimized, ensuring proportionality of benefits and harms in all decisions.

14. Relevance *

Select only one option.

Not relevant

Somewhat relevant

Quite relevant

Highly relevant

15. Clarity *

Select only one option

Not clear at all

Somewhat clear

Quite clear

Highly clear

16. Simplicity *

Select only one option.

Not simple

Somewhat simple

Quite simple

Highly simple

17. Applicability *

Select only one option.

Not applicable

Somewhat applicable

Quite applicable

Highly applicable

18. Comments

Fundamental rights and principles

4 - Respect for autonomy and individual responsibility

The autonomy of individuals to make and take responsibility for their decisions, while respecting others' autonomy, must be upheld and promoted. In dementia care, the progressive cognitive decline reduces autonomy, necessitating tailored measures to protect the rights and interests of those affected. Promoting autonomy means fostering relationships, offering meaningful choices in daily activities, and supporting individuals in maintaining their identity and values, even as decision-making abilities decline. When individuals can no longer exercise autonomy, trusted people may need to make decisions on their behalf, guided by advanced directives, known preferences, or consistent behaviors, in line with their rights and dignity.

19. Relevance *

Select only one option.

Not relevant

Somewhat relevant

Quite relevant

Highly relevant

20. Clarity *

Select only one option.

Not clear at all

Somewhat clear

Quite clear

Highly clear

21. Simplicity *

Select only one option.

Not simple

Somewhat simple

Quite simple

Highly simple

22. Applicability *

Select only one option.

Not applicable

Somewhat applicable

Quite applicable

Highly applicable

23. Comments

Informed consent and capacity

*5 - **Consent***

Consent is a process based on a trusted relationship between patient and health and social care professionals. Any preventive, diagnostic and therapeutic intervention must be carried out with the prior, free and informed consent of the PwD. This consent must be based on adequate and truthful information provided within comprehensible communication and should be obtained in the manner and by the most appropriate means to PwD, who can also involve family members and/or trusted persons to make joint decisions.

The ability of PwD to give (and withdraw) consent should not be underestimated and should be carefully assessed as the diagnosis of dementia and/or neuropsychological tests performance do not identify competence, which is task-specific and may vary over time.

Advance care planning should be promoted for PwD to communicate their own idea of what they value and to communicate their choices before the severe stage of the disease prevents it.

24. Relevance *

Select only one option.

Not relevant

Somewhat relevant

Quite relevant

Highly relevant

25. Clarity *

Select only one option.

Not clear at all

Somewhat clear

Quite clear

Highly clear

26. Simplicity *

Select only one option.

Not simple

Somewhat simple

Quite simple

Highly simple

27. Applicability *

Select only one option.

Not applicable

Somewhat applicable

Quite applicable

Highly applicable

28. Comments

Informed consent and capacity

6 - ***Persons without the capacity to consent***

Lack of capacity to give consent cannot be a reason to deny PwD the best possible health and social intervention. In accordance with domestic law, special protection is to be given to PwD who do not have the capacity to consent.

Decisions for health and social intervention should be aligned with what the individual would have wanted based on their advanced directives, past statements, or consistent behaviors and beliefs; when an individual's preferences are unknown decisions should be taken in accordance with their best interest.

The PwD should be involved to the greatest extent possible in the decision-making process; their assent should be sought whenever possible and their refusal considered.

29. Relevance *

Select only one option.

Not relevant

Somewhat relevant

Quite relevant

Highly relevant

30. Clarity *

Select only one option.

- Not clear at all
- Somewhat clear
- Quite clear
- Highly clear

31. Simplicity *

Select only one option.

- Not simple
- Somewhat simple
- Quite simple
- Highly simple

32. Applicability *

Select only one option.

- Not applicable
- Somewhat applicable
- Quite applicable
- Highly applicable

33. Comments

Respect for individuality and privacy

*7 - **Respect for individual identity, personal integrity and human vulnerability***

In applying and advancing scientific knowledge, clinical practice and technologies, individual identity, personal integrity and human vulnerability should be considered.
The PwD remains the same, equally valued, person throughout the course of their illness, regardless of the extent of the changes in their cognitive and other functions: their identity should be recognized and their personal integrity respected.
Respect for personal integrity is respect for the physical and mental life of the individual, for their own understanding of their life and illness and for their life-story.
The PwD can be in a position of increased vulnerability, as their dignity or autonomy or integrity may be threatened: they should be protected to the necessary extent and receive adequate support to enable them to realize their potential.

34. Relevance *

Select only one option.

Not relevant

Somewhat relevant

Quite relevant

Highly relevant

35. Clarity *

Select only one option.

Not clear at all

Somewhat clear

Quite clear

Highly clear

36. Simplicity *

Select only one option.

Not simple

Somewhat simple

Quite simple

Highly simple

37. Applicability *

Select only one option.

Not applicable

Somewhat applicable

Quite applicable

Highly applicable

38. Comments

Respect for individuality and privacy

*8 - **Privacy and confidentiality***

Respect for personal integrity entails that the privacy of PwD and the confidentiality of their personal information should be respected. Such information should not be used or disclosed for purposes other than those for which it was collected or consented to, consistent with domestic and international law.

39. Relevance *

Select only one option.

Not relevant

Somewhat relevant

Quite relevant

Highly relevant

40. Clarity *

Select only one option.

Not clear at all

Somewhat clear

Quite clear

Highly clear

41. Simplicity *

Select only one option.

Not simple

Somewhat simple

Quite simple

Highly simple

42. Applicability *

Select only one option.

Not applicable

Somewhat applicable

Quite applicable

Highly applicable

43. Comments

Equality, inclusion, and non-discrimination

*9 - **Equality, justice, and equity***

The inherent dignity and fundamental rights of all human beings must be upheld in dementia care, in a just and equitable way. Equality ensures that all PwD are treated with equal respect, and access to care and support. Justice requires fair access to care and resources, while equity demands that care be tailored to the unique needs of each person, recognizing their specific circumstances and challenges.

44. Relevance *

Select only one option.

Not relevant

Somewhat relevant

Quite relevant

Highly relevant

45. Clarity *

Select only one option.

Not clear at all

Somewhat clear

Quite clear

Highly clear

46. Simplicity *

Select only one option.

Not simple

Somewhat simple

Quite simple

Highly simple

47. Applicability *

Select only one option.

Not applicable

Somewhat applicable

Quite applicable

Highly applicable

48. Comments

Equality, inclusion, and non-discrimination

*10 - **Solidarity***

Solidarity in dementia care involves a collective responsibility to support people with dementia. It calls for fostering inclusive and adapted environments and attitudes that combat stigma, while promoting the dignity and rights of those affected by dementia.

49. Relevance *

Select only one option.

Not relevant

Somewhat relevant

Quite relevant

Highly relevant

50. Clarity *

Select only one option.

Not clear at all

Somewhat clear

Quite clear

Highly clear

51. Simplicity *

Select only one option.

Not simple

Somewhat simple

Quite simple

Highly simple

52. Applicability *

Select only one option.

Not applicable

Somewhat applicable

Quite applicable

Highly applicable

53. Comments

Equality, inclusion, and non-discrimination

*11 - **Non-discrimination and non-stigmatization***

PwD must be protected from all forms of discrimination and stigma. Discrimination arises from unequal access to healthcare, social services, or employment based on characteristics such as race, ethnicity, gender, age, disability, sexual orientation, or other personal attributes. Stigmatization stems from biases, negative stereotypes, and misconceptions that marginalize and dehumanize PwD. Both practices undermine human dignity, rights, and freedoms, creating barriers to essential care and support.

54. Relevance *

Select only one option.

Not relevant

Somewhat relevant

Quite relevant

Highly relevant

55. Clarity *

Select only one option.

Not clear at all

Somewhat clear

Quite clear

Highly clear

56. Simplicity *

Select only one option.

Not simple

Somewhat simple

Quite simple

Highly simple

57. Applicability *

Select only one option.

Not applicable

Somewhat applicable

Quite applicable

Highly applicable

58. Comments

Equality, inclusion, and non-discrimination

*12 - **Respect for cultural diversity and pluralism***

Dementia care must respect cultural diversity and pluralism by recognizing and valuing the distinct cultural, linguistic, and social backgrounds of all people with dementia. Care approaches should be sensitive to these differences while ensuring that cultural practices do not compromise human dignity, rights, or freedoms. While cultural diversity enriches care, all practices must align with the overarching principles of dignity, rights, and equity, enhancing—rather than limiting—the quality and accessibility of care.

59. Relevance *

Select only one option.

Not relevant

Somewhat relevant

Quite relevant

Highly relevant

60. Clarity *

Select only one option.

Not clear at all

Somewhat clear

Quite clear

Highly clear

61. Simplicity *

Select only one option.

Not simple

Somewhat simple

Quite simple

Highly simple

62. Applicability *

Select only one option.

Not applicable

Somewhat applicable

Quite applicable

Highly applicable

63. Comments

Rights and responsibilities

*13 – **Citizens´ rights, political and democratic participation***

According to their capacity, PwD have the right to participate in political processes and contribute to decisions that affect them, at both the national and local levels. All PwD have the right to freedom of expression.

.

64. Relevance *

Select only one option.

Not relevant

Somewhat relevant

Quite relevant

Highly relevant

65. Clarity *

Select only one option.

Not clear at all

Somewhat clear

Quite clear

Highly clear

66. Simplicity *

Select only one option.

Not simple

Somewhat simple

Quite simple

Highly simple

67. Applicability *

Select only one option.

Not applicable

Somewhat applicable

Quite applicable

Highly applicable

68. Comments

Rights and responsibilities

*14 - **Right to health and social care***

People living with dementia have rights to equitable access to high quality medical and social care, opportunities to maintain social health, be integrated in the social community and continue participation in social life to the desired. Interactions with people with dementia should be grounded in empirical evidence, relevant professional standards, cultural competence and communication skills to ensure the highest standards of care.

69. Relevance *

Select only one option.

Not relevant

Somewhat relevant

Quite relevant

Highly relevant

70. Clarity *

Select only one option.

Not clear at all

Somewhat clear

Quite clear

Highly clear

71. Simplicity *

Select only one option.

Not simple

Somewhat simple

Quite simple

Highly simple

72. Applicability *

Select only one option.

Not applicable

Somewhat applicable

Quite applicable

Highly applicable

73. Comments

Rights and responsibilities

*15 - **Training and skills development***

Professionals involved in dementia care must receive adequate training and education to provide high-quality care. The quality of care provided shall be measured by adequate indicators and monitored regularly, being a continuous dynamic and iterative process of planning, doing, checking and acting.

74. Relevance *

Select only one option.

Not relevant

Somewhat relevant

Quite relevant

Highly relevant

75. Clarity *

Select only one option.

Not clear at all

Somewhat clear

Quite clear

Highly clear

76. Simplicity *

Select only one option.

Not simple

Somewhat simple

Quite simple

Highly simple

77. Applicability *

Select only one option.

Not applicable

Somewhat applicable

Quite applicable

Highly applicable

78. Comments

Rights and responsibilities

16 - ***Public responsibility***

Public health professionals have the responsibility to support caregivers and relatives in their roles through education and respite from their duties and to educate and inform general society of the emerging risk reduction strategies to reduce dementia prevalence and incidence.

79. Relevance *

Select only one option.

Not relevant

Somewhat relevant

Quite relevant

Highly relevant

80. Clarity *

Select only one option.

Not clear at all

Somewhat clear

Quite clear

Highly clear

81. Simplicity *

Select only one option.

Not simple

Somewhat simple

Quite simple

Highly simple

82. Applicability *

Select only one option.

Not applicable

Somewhat applicable

Quite applicable

Highly applicable

83. Comments

Please respond to the following three questions based on your overall appraisal of the Framework, and kindly provide a brief comment for each item.

84. To what extent do you think the framework will be useful to guide your ethical decisions in daily practice? *

Select only one option.

Not useful

Somewhat useful

Quite useful

Highly useful

85. Comments

86. To what extent do you think the framework sufficiently covers all aspects of dementia care ethics? *

Select only one option.

Not sufficient

Somewhat sufficient

Quite sufficient

Highly sufficient

87. Comments

88. Do you feel motivated to start using this framework when it will be released and to disseminate it among your peers? *”
